# Dengue Fever Occurrence and Vector Detection by Larval Survey, Ovitrap and MosquiTRAP: A Space-Time Clusters Analysis

**DOI:** 10.1371/journal.pone.0042125

**Published:** 2012-07-25

**Authors:** Diogo Portella Ornelas de Melo, Luciano Rios Scherrer, Álvaro Eduardo Eiras

**Affiliations:** 1 Lab. Ecologia Química de Insetos Vetores, Departamento de Parasitologia, Instituto de Ciências Biológicas, Universidade Federal de Minas Gerais. Av. Presidente Antônio Carlos, Belo Horizonte, MG, Brazil; 2 Departamento de Estatística, Instituto de Ciências Exatas, Universidade Federal de Minas Gerais, Belo Horizonte, MG, Brazil; New Mexico State University, United States of America

## Abstract

The use of vector surveillance tools for preventing dengue disease requires fine assessment of risk, in order to improve vector control activities. Nevertheless, the thresholds between vector detection and dengue fever occurrence are currently not well established. In Belo Horizonte (Minas Gerais, Brazil), dengue has been endemic for several years. From January 2007 to June 2008, the dengue vector *Aedes (Stegomyia) aegypti* was monitored by ovitrap, the sticky-trap MosquiTRAP™ and larval surveys in an study area in Belo Horizonte. Using a space-time scan for clusters detection implemented in SaTScan software, the vector presence recorded by the different monitoring methods was evaluated. Clusters of vectors and dengue fever were detected. It was verified that ovitrap and MosquiTRAP vector detection methods predicted dengue occurrence better than larval survey, both spatially and temporally. MosquiTRAP and ovitrap presented similar results of space-time intersections to dengue fever clusters. Nevertheless ovitrap clusters presented longer duration periods than MosquiTRAP ones, less acuratelly signalizing the dengue risk areas, since the detection of vector clusters during most of the study period was not necessarily correlated to dengue fever occurrence. It was verified that ovitrap clusters occurred more than 200 days (values ranged from 97.0±35.35 to 283.0±168.4 days) before dengue fever clusters, whereas MosquiTRAP clusters preceded dengue fever clusters by approximately 80 days (values ranged from 65.5±58.7 to 94.0±14. 3 days), the former showing to be more temporally precise. Thus, in the present cluster analysis study MosquiTRAP presented superior results for signaling dengue transmission risks both geographically and temporally. Since early detection is crucial for planning and deploying effective preventions, MosquiTRAP showed to be a reliable tool and this method provides groundwork for the development of even more precise tools.

## Introduction

The Dengue fever is an arboviral disease with a wide geographical range and high case numbers. Annually 50 million infections are estimated, 500,000 cases progress to dengue hemorrhagic fever (DHF), and there are more than 20,000 deaths per year [1]. Dengue viruses (Flaviviridae) are transmitted to humans by certain species of mosquitoes (Diptera: Culicidae) belonging to the genus *Aedes*. *Aedes (Stegomyia) aegypti* is the main vector worldwide, and is present in urban environments. *Aedes (Stegomyia) albopictus*, is currently considered of secondary importance in transmission, except in Asian countries, being present in rural or semi-urban habitats [2]. In the Americas, where the species probably arrived by passive transport during slave trade, *Ae. aegypti* is commonly found inside human dwellings where it can both rest and obtain blood meals [3]–[4].

Belo Horizonte city (Minas Gerais, Brazil. 19°49′13′′S; 43°55′06′′W) has approximately 2.5 million inhabitants and 330.9 km^2^ of area [5]. In the last two decades, the city has recorded endemic cases of dengue fever. [6]–[9].

In Belo Horizonte epidemiological surveillance and vector control is implemented according to the guidelines of Programa Nacional de Controle da Dengue (PNCD), including routine entomological surveillance and control of immature life stages [10]. Entomological surveillance is carried out as larval surveys using the following methodologies: 1) surveys of Strategic Points (SP)-periodical inspections of premises that have large numbers of reservoirs serving as breeding site for mosquitoes, such as tire stores and junkyards; 2) *Levantamento Rápido de Índice de Infestação (*LIR*Aa)*-sampled survey of premises, held three times yearly; 3) Special Vector Surveys (SVS)-any survey held in premises for detection of immatures that extrapolates previous cited methods (e.g., in areas where dengue fever cases occurred, where vectors were detected by other monitoring methods, or by citizens complaint) [10]–[12]. However, no association was observed between household infestation index values and risk of dengue infection in many Brazilian cities [13].

Oviposition traps, ovitraps [14] were routinely used in Belo Horizonte since 2002. The ovitraps filled with a 10% dilution of *Panicum maximum* infusion prepared at seven days were installed by vector control personnel at each point individually. Ovitrap placement occurred between periods of 14 days, and were inspected after 7 days, after which each trap was removed and its paddle was sent to the municipality’s entomology laboratory for egg counting [15].

**Figure 1:**
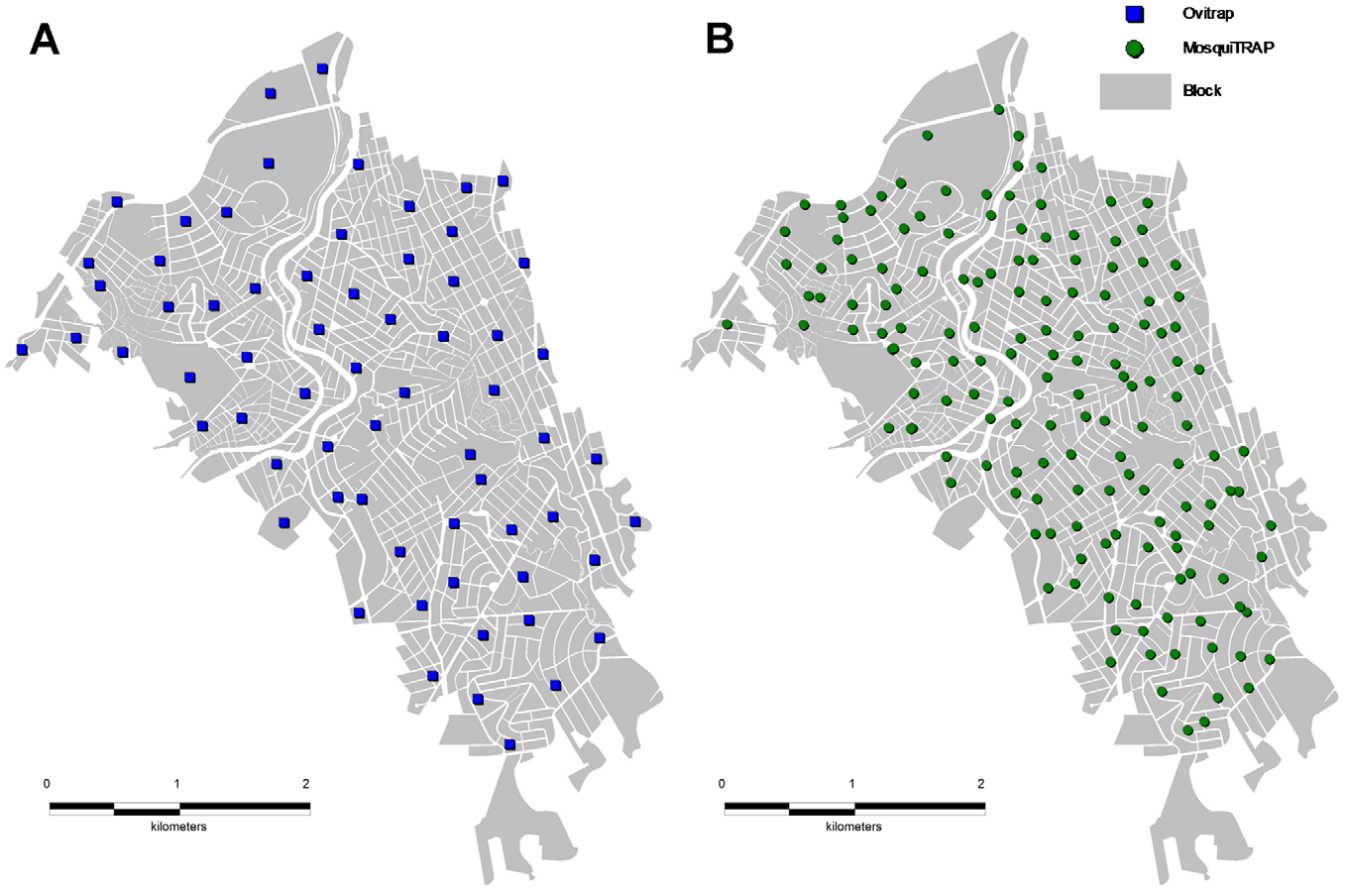
Ovitrap (A) and MosquiTRAP (B) deployment sites. Ovitrap deployment sites are represented in blue and MosquiTRAP sites in dark green.

Recently, a new methodology namely MI-Dengue™ (*Computerized System for Intelligent Dengue Monitoring)* has become available for large scale monitoring of dengue vectors. MI-Dengue includes a trap (MosquiTRAP™) that catches gravid females of *Ae. aegypti* and a computerized system that allows for rapid generation of *Aedes* infestation maps. The MosquiTRAP includes an adhesive, sticky card inside the trap and allows for species identification during trap inspection by vector control personnel [16]–[18]. Mosquitrap is additionally advantageous over other larval survey methods since time spent at residences is reduced, implying cost reduction and fast data collection [19]. The MosquiTRAP has been compared with larval surveys and ovitraps [20]–[22], and also used to determine temporal patterns of *Ae. aegypti* population dynamics [23], in mark-release-recapture studies [24] and for development of a new entomological index for dengue vector control [17]. MosquiTRAP showed to be more sensitive than larval survey [18], [19], [23] and equally [20] or less sensitive than ovitrap [23], for detection and monitoring of *Ae. aegypti* in urban areas.

Spatial analysis of health events contributes to early detection of situations involving disease transmission, and aids epidemiological surveillance services in decisions and in the evaluation of risk factors concerning infectious or noninfectious diseases. The detection of disease clusters allows the identification of non-random events, and inferences about their epidemiological determinants [25]. Space-time scan statistics, a methodology implemented in the software SaTScan by Kulldorff, enables the detection of clusters that depend on spatial and temporal relationships of events [26]–[29]. For determination of factors affecting the distribution of events, space-time cluster detection is a more accurate technique compared to purely spatial scan, as it assesses the two dimensions simultaneously, avoiding multiple tests bias [30]. The scale at which the data will be aggregated is an important consideration for the detection of clusters. Aggregation units often used in scan statistics are the exact coordinates, zip-codes, blocks, census tracts, districts, cities or states [31], [32]. Space time scan statistics have been applied in a variety of studies, including non-infectious diseases [28], [33], [34], and infectious diseases such as malaria [35]–[37], West Nile virus encephalitis [38], [39], African trypanosomiasis [40] and dengue [41], [42].

**Figure 2:**
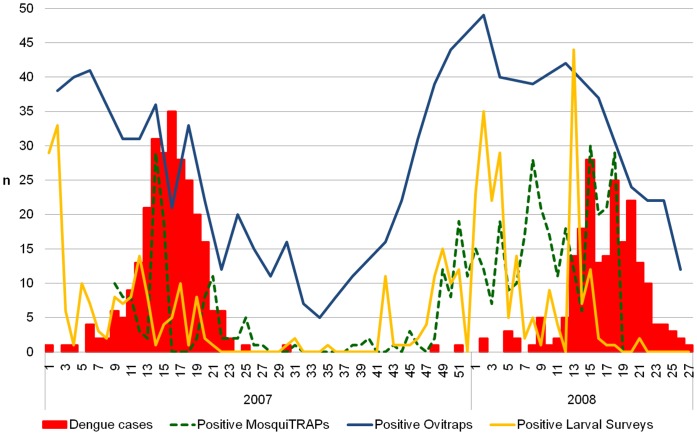
Distribution of collected data along the study period. The graphic shows the number of registries by week for each dataset, dengue fever cases (red), positive MosquiTRAPs (dash green), positive ovitraps (blue) and positive larval surveys (yellow).

Despite the knowledge of *Ae. aegypti* biology and large-scale use of monitoring tools by epidemiological surveillance and vector control services, the correlation between the detection of the vector and the occurrence of the disease remains poorly understood [43]. The aim of this study was to evaluate how different methods of *Ae. aegypti* surveillance correlates to dengue fever occurrence on a pre-selected area of Belo Horizonte from 2007–2008.

## Methodology

### Ethics Statement

The access to patients data, including the home adress, was approved by Ethics Committee of Secretaria Municipal de Saúde de Belo Horizonte (SMSA-PBH).

### Study Area and Data Source

The study area (19°57′04′′S;43°58′56′′W) was a pre-seleted urban area in Belo Horizonte (Minas Gerais, Brazil) where the monithoring of *Ae. aegypti* was performed simultaneously using larval survey, ovitrap and MosquiTRAP and comprised a total of 13.808 Km^2^. The study period was from January 1^st^, 2007 to June 30^th^, 2008. Dengue fever registries and vector surveillance data by larval survey, ovitrap and MosquiTRAP were obtained from the Secretaria Municipal de Saúde de Belo Horizonte (SMSA-PBH). The geographic data of each block-location, area and population was obtained from the SMSA-PBH and from the current demographic census [5].

**Table 1:** Ovitrap-annual results of *Aedes sp.* monitoring. 2007–2008.

Year	Inspected	Positive	OPI^1^	Eggs collected	EDI^2^	s.d
2007	1414	550	38.90	29792	54.17	64.56
2008	570	287	50.35	15023	52.34	61.50
*Total*	*1984*	*837*	*42.19*	*44815*	*53.54*	*63.49*

1 OPI  =  Ovitrap Positivity Index;

2 EDI  =  Eggs Density Index.

**Table 2:** MosquiTRAP-annual results of *Aedes aegypti* monitoring. 2007–2008.

Year	Inspected	Positive	MPI^1^	Females captured	MFAI^2^	s.d.
2007	3995	175	4.38	314	0.08	0.49
2008	1779	302	16.97	499	0.28	0.79
*Total*	*5774*	*477*	*8.26*	*813*	*0.14*	*0.61*

1 MPI  =  MosquiTRAP Positivity Index;

2 MFAI  =  Mean Female Aedes Index.

### Data Acquisition

#### Dengue fever cases

Only autochthonous confirmed dengue fever cases were considered. Records were geoprocessed using the municipality database comprising the geographic coordinates (metric system) of each recorded patient address. The coordinate system used was the Universal Transverse Mercator (UTM) zone 23°, SAD69 datum. If exact coordinates were unable to be obtained, an approximate address coordinate was considered. Records whose coordinates were not obtained were ignored. The records were grouped by date of dengue symptoms onset and the patient’s home address block. The criterion for confirmation of dengue fever was not considered in this study, cases confirmed by laboratory tests or by clinical and epidemiological criterion were not distinguished.

**Table 3:** Larval survey-annual results of *Aedes aegypti* monitoring by year and category. 2007–2008.

Category of survey	2007		2008		Total surveyed	Total positive	Positivity (%)
	Surveyedpremises	Positivepremises	Surveyedpremises	Positivepremises			
LIR*Aa* 1	5971	76	4992	79	10963	155	1.41
SP^2^	959	20	544	25	1503	45	2.99
SVS^3^	317	144	224	114	541	258	47.69
*Total*	*7247*	*240*	*5760*	*218*	*13007*	*458*	*3.52*

1 LIRAa  =  Levantamento rápido de índice para *Aedes aegypti*;

2 SP  =  Strategic Points Survey;

3 SVS  =  Special Vector Survey.

**Figure 3:**
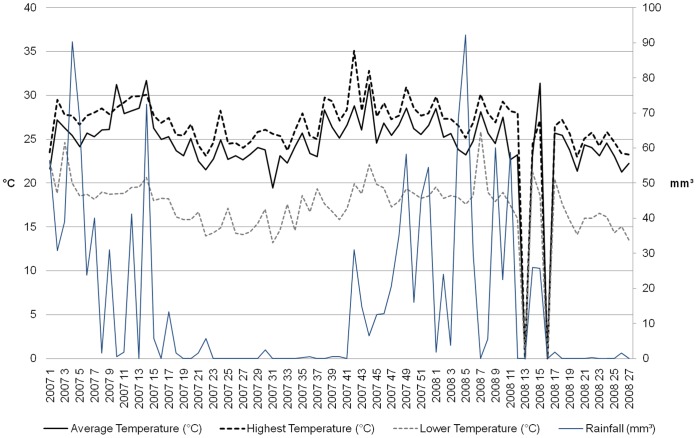
Meteorological data of Belo Horizonte, Minas Gerais, Brazil, January 1^st^, 2007 to June 30^th^, 2008. Demonstrates two different seasons, a warmer and umid summer and a dry winter with mild temperatures. Average Temperature: 25,17±3,66°C (black), Highest Temperature: 27,08±3,17°C (dash black); Lower Temperature: 17,74±4,01°C (dash gray); Precipitation average: 2,41±7,03 mm^3^ (blue). *Source: CPTEC/INPE*.

#### Larval survey

Larval surveys were conducted by vector control personnel following the PNCD guideline and consisted in premises inspection for larvae detection [10]–[12]. The categories Strategic Points inspection (SP), Special Vectors Survey (SVS) and LIR*Aa* were considered [12]. The data were obtained from SMSA-PBH and contained the date of premises inspection, inspected premises block identification number, the presence or absence of larvae and the species identification which was executed by the municipality’s laboratory.

**Table 4:** Summary of dengue fever space-time clusters detected using different MCS values.

MCS^1^	Clusters	Radius (m)		Area (km^2^)			Length (days)		
		Average	s.d.	Sum	Average	s.d.	Sum	Average	s.d.
200 m	16	160.768	37.537	1.293	0.081	0.054	826	51.625	24.336
300 m	12	223.503	72.435	1.677	0.140	0.083	525	43.750	28.974
400 m	8	279.859	105.856	1.763	0.220	0.148	425	53.125	31.009
500 m	7	355.116	149.689	2.473	0.353	0.215	305	43.571	24.213
600 m	5	552.798	27.467	3.692	0.738	0.127	249	49.800	14.412
700 m	4	618.182	62.260	3.539	0.885	0.238	235	58.750	10.813
800 m	4	682.116	66.294	3.648	0.912	0.321	233	58.250	10.905

1 MCS  =  Maximum Cluster Size.

#### Ovitrap

Traps were installed and inspected every 15 days by vector control personnel and geolocated at the deployment block coordinates (**Figure 1A**). The records of vector monitoring specified the number of *Aedes* sp. eggs obtained from each period of trap deployment for each trap. The species identification was not considered.

**Table 5:** Summary of positive ovitraps space-time clusters detected using different MCS values.

MCS^1^	Clusters	Radius (m)		Area (km^2^)			Length (days)		
		Average	s.d.	Sum	Average	s.d.	Sum	Average	s.d.
200 m	5	27.184	60.786	0.598	0.120	0.208	1290	258.000	88.544
300 m	6	183.447	102.362	1.150	0.192	0.155	1142	190.333	76.894
400 m	8	274.208	133.603	2.423	0.303	0.175	1448	181.000	72.938
500 m	8	338.279	165.192	3.185	0.398	0.161	1336	167.000	81.290
600 m	7	386.961	209.684	3.520	0.503	0.227	1330	190.000	69.733
700 m	7	435.159	248.166	4.339	0.620	0.369	1218	174.000	85.159
800 m	5	438.635	352.170	4.049	0.810	0.611	1010	202.000	79.812

1 MCS  =  Maximum Cluster Size.

#### MosquiTRAP

The sticky trap MosquiTRAP™ consists of a matte black container (33cm high by 15cm wide) filled with approximately 300 ml of tap water, and an odorless sticky card that holds the captured mosquitoes and is attached from the water line [17]. A synthetic oviposition attractant (AtrAedes™) was attached to the sticky card. The traps were placed at maximum height of 1.5 m above ground, sheltered from sun and rain, out of reach of domestic animals and children. Mosquitoes were identified using a magnifying glass (20x) during trap inspection, recorded, and removed from the sticky card. The records were geolocated by block of trap deployment (**Figure 1B**) and specified the number of female *Ae. aegypti* collected in each trap during the monitored week. MosquiTRAPs were placed during the 2^nd^ week of 2007 and removed on the 18^th^ week 2008. The sticky traps were inspected weekly, but no inspections were conducted in 2007 on weeks 16, 17, 32–35. Data were collected by an electronic spreadsheet installed in palmtop computers (Model 515, Palmtop, USA) and processed using the MI-Dengue computerized system.

**Table 6:** Summary of positive MosquiTRAPs space-time clusters detected using different MCS values.

MCS^1^	Clusters	Radius (m)		Area (km^2^)			Length (days)		
		Average	s.d.	Sum	Average	s.d.	Sum	Average	s.d.
200 m	6	92.612	101.790	0.465	0.077	0.092	491	81.833	32.053
300 m	7	256.358	53.950	1.349	0.193	0.085	623	89.000	34.157
400 m	6	343.289	82.538	2.051	0.342	0.145	512	85.333	38.764
500 m	6	360.814	96.625	2.283	0.381	0.171	505	84.167	38.701
600 m	6	455.767	154.386	3.408	0.568	0.300	519	86.500	52.523
700 m	5	531.372	207.068	3.965	0.793	0.431	478	95.600	50.816
800 m	5	586.424	235.742	5.222	1.044	0.596	478	95.600	42.117

1 MCS  =  Maximum Cluster Size.

**Table 7:** Summary of space-time clusters of positive larval survey premises detected using different MCS values.

MCS^1^	Clusters	Radius (m)		Area (km^2^)			Length (days)		
		Average	s.d.	Sum	Average	s.d.	Sum	Average	s.d.
200 m	9	165.693	24.834	0.713	0.079	0.024	349	38.778	33.607
300 m	6	252.546	55.286	1.062	0.177	0.094	242	40.333	27.038
400 m	5	372.897	26.110	1.855	0.371	0.040	169	33.800	21.534
500 m	5	444.086	74.411	2.775	0.555	0.211	179	35.800	27.617
600 m	3	566.590	33.168	2.204	0.735	0.083	137	45.667	20.257
700 m	3	623.935	117.341	2.223	0.741	0.303	119	39.667	26.083
800 m	3	778.257	23.387	3.662	1.221	0.386	80	26.667	31.628

1 MCS  =  Maximum Cluster Size.

The MI-Dengue system consists of recording field data from the MosquiTRAP with an electronic spreadsheet and specific software (Geo-Dengue, Ecovec Ltda, Belo Horizonte, Brazil). This system allows municipal health managers to access and view information on the density of gravid female *Ae. aegypti* on georeferenced maps and in analytical tables of the sites monitored with MosquiTRAP. The data acquisition on electronic spreadsheets was acquired daily during trap inspection by means of an electronic spreadsheet and the Geo-Dengue software. These data were transferred automatically to the municipality’s database, and the site automatically generated the georeferenced maps and tables for the municipal manager. The electronic spreadsheet recorded the household data (e.g., resident’s name, address, ZIP code, and place where trap was installed in the residence), data on adult mosquitoes captured and their respective numbers per trap installed in the residences for each block in the monitored municipalities, thus facilitating the work by the field inspector during trap inspection. Field inspectors were trained to identify the mosquitoes captured in the MosquiTRAP with the aid of a field magnifying glass (20x), where they generally identified *Ae. aegypti* and *Ae. albopictus* by species and sex, recorded the number of specimens captured on the electronic spreadsheet. For mosquitoes from the genus *Culex* and other *Aedes (non-aegypti)* species, only the genus-level information was recorded.

**Figure 4:**
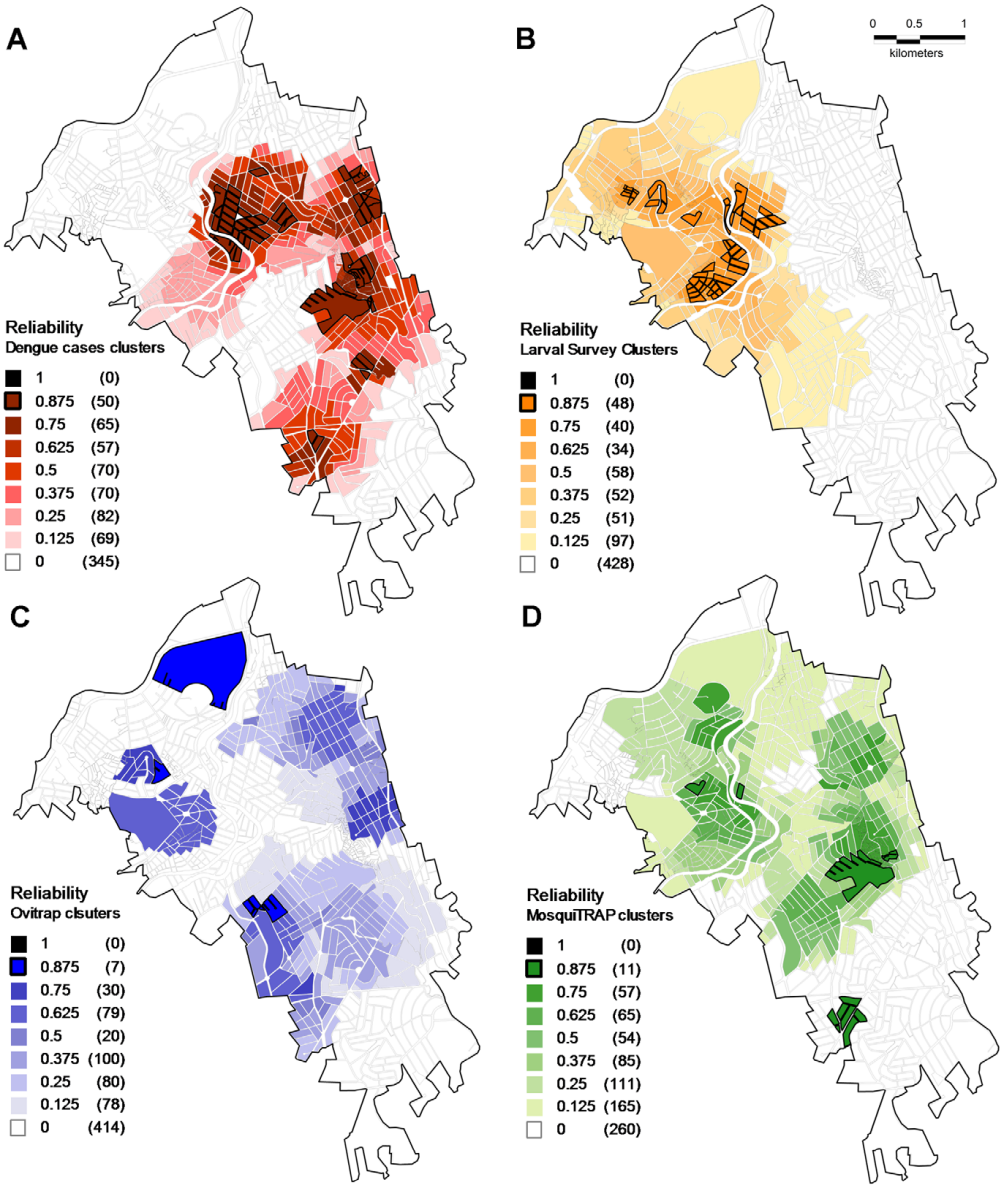
Clusters reliability maps. **A**: Dengue fever clusters; **B**: Positive larval survey clusters; **C**: Positive ovitrap clusters Positive; **D**: MosquiTRAP clusters. Dark colors represents higher reliability values (*Ri*).

**Table 8:** Clusters reliability evaluation.

Dataset	*Reliability Values (Ri)*							
	0.125	0.25	0.375	0.5	0.625	0.75	0.875	Total
Dengue Fever	69	82	70	70	57	65	50	463
	0.880	0.794	0.785	0.756	0.464	0.518	0.688	4.884
MosquiTRAP	242	114	85	54	65	57	11	628
	2.999	1.291	0.656	0.645	0.646	0.547	0.300	7.084
Ovitrap	78	80	100	20	79	30	7	394
	1.091	1.087	1.133	0.198	1.176	0.314	0.578	5.576
Larval survey	98	51	52	58	34	40	48	381
	1.749	0.718	0.813	0.750	0.288	0.349	0.333	5.001

The table contains the number of blocks that presented each *Ri* value and the total area of the blocks (km^2^). For each registry, the upper line contains the number of blocks (in units) and the lower line the sum of blocks area (km^2^).

### Space-time Cluster Detection

Clusters of high incidence of dengue fever and high occurrence of *Ae. aegypti* were detected using Space-Time Scan Statistics [28] implemented in SaTScan® v.8.1. The space-time scan statistic uses a cylindrical window (the base is geographic and the height corresponds to time). The windows moves in space and time, considering each geographic location and each time interval, so that a infinite number of overlapping cylinders is set throughout the study region. The null hypothesis assums that events are distributed ramdomly, and with different risk inside and outside in at least one clylinder under alternative hypothesis. So, each cylinder is a possible cluster, and considering the number of events inside and outside and an expected number of events (calculated based in population at risk and/or covariates), the likelihood is calculated. The cylinder with maximun likelihood and an excess of number of events relative to the expected, is denoted as the most likely cluster [28].

**Figure 5:**
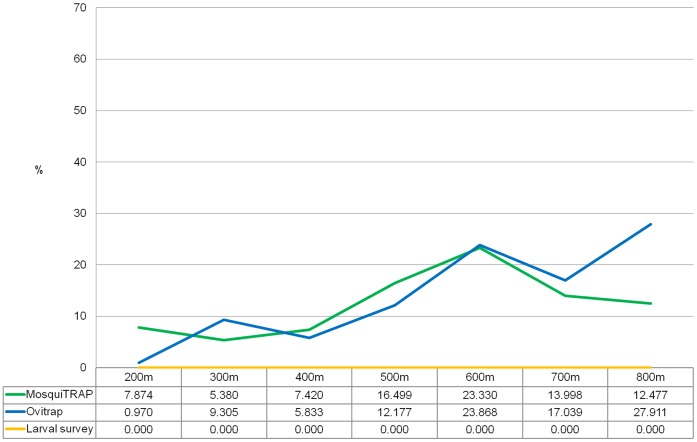
Spatial intersection of *Ae. aegypti* clusters to dengue fever clusters. Considering different monitoring methods and different values of Maximum Clusters Size parameter (MCS). MosquiTRAP (green), ovitrap (blue) and larval survey (yellow).

**Figure 6:**
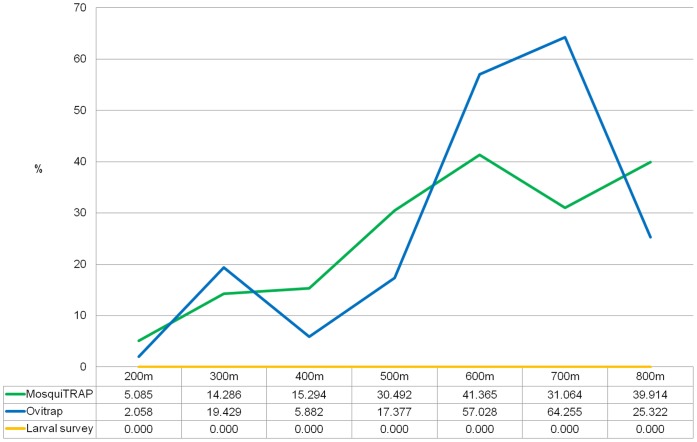
Temporal intersection of *Ae. aegypti* clusters to dengue fever clusters. Considering different monitoring methods and different values of Maximum Clusters Size parameter (MCS). MosquiTRAP (green), ovitrap (blue) and larval survey (yellow).

**Figure 7:**
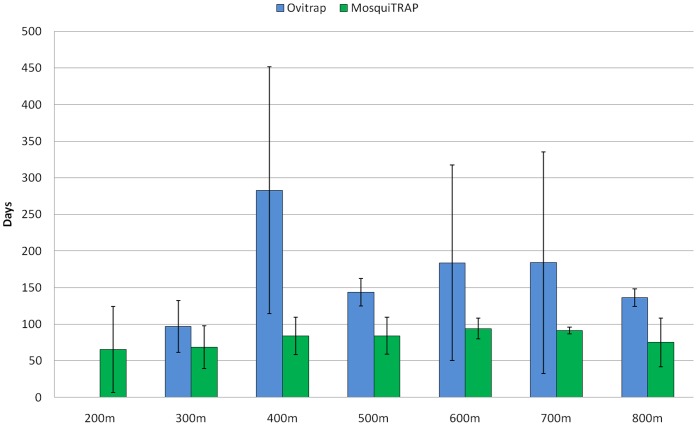
Average days that *Ae. aegypti* clusters preceded the occorrence of dengue fever clusters in spatially overlapped clusters areas. Comparision of ovitrap (dark gray) and MosquiTRAP (light gray) methods considering different values of Maximum Clusters Size parameter (MCS).

**Table 9:** Summary of temporal intersections of *Ae. aegypti* clusters (MosquiTRAP detected) to dengue fever clusters.

MCS^1^	Temporal intersections			Precedence		
	n	Mean	s.d.	n	Mean	s.d.
200 m	2	21.00	4.24	2	65.50	58.69
300 m	4	18.75	15.56	4	68.50	29.15
400 m	2	32.50	17.68	2	84.00	25.46
500 m	4	23.25	12.26	4	84.25	25.02
600 m	4	25.75	11.84	5	94.00	14.30
700 m	3	24.33	12.42	3	91.33	4.62
800 m	3	31.00	1.73	3	75.33	33.23

The table also contains the average period (days) of vector clusters precedence related to spatially overlapped dengue fever clusters.

1 MCS  =  Maximum Cluster Size.

**Table 10:** Summary of temporal intersections of *Aedes sp.* eggs clusters (ovitrap detected) to dengue fever clusters.

MCS^1^	Temporal intersections			Precedence		
	n	Mean	s.d.	n	Mean	s.d.
200 m	1	17.00	0	1	40.00	0
300 m	2	51.00	1.41	2	97.00	35.36
400 m	2	12.50	10.61	4	283.00	168.40
500 m	2	26.50	26.50	4	143.75	18.73
600 m	4	35.50	16.62	6	183.83	133.41
700 m	3	50.33	10.12	5	184.00	151.32
800 m	2	29.50	20.51	2	136.50	12.02

The table also contains the average period (days) of vector clusters precedence related to spatially overlapped dengue fever clusters.

1 MCS  =  Maximum Cluster Size.

#### Dengue fever clusters

The space-time scan statistic was implemented using space-time retrospective analyses to scan for areas with high rates of dengue incidence. The analyses were carried out using the Poisson probability model, with 999 Monte Carlo replications to test for significance, and not allowing geographic overlapping clusters. The time aggregation unit was the day, and the maximum temporal cluster size used was the software default, 50% of the study period. The number of dengue fever cases by block was used as the case file. The start date of symptoms was used to determine the date of a dengue fever case. Population size for each block was estimated from census data and used as the population file. The latitude and longitude of the centroids of each block were used as the block spatial coordinates. The default setting of 50% of the population at risk used by SaTScan seldom produces usable, informative results because the reported primary cluster often occupies a large proportion of the study area, hiding other small clusters that may be detected and important at a regional scale [44]–[49]. Thus, we used a range of Maximum Cluster Sizes (MCS), including 200, 300, 400, 500, 600, 700 and 800 m.

#### *Aedes aegypti* clusters

Analyses were carried out using the Bernoulli probability model to scan for areas with high rates of positive traps or positive surveyed premises. The analyses were performed using 999 Monte Carlo replications to test for significance, with no geographical overlap allowed. For larval survey data, time aggregation unit was the day. For traps monitoring data, the time aggregation unit was the week (7 day period), since the traps were inspected weekly (MosquiTRAPs) or biweekly (ovitraps). The maximum temporal cluster size was the software default, 50% of study period. Positive larval survey premises were associated with blocks of occurrence and survey date in the case file. Negative larval survey premises comprised the control file. For the traps datasets, positive traps were associated with blocks and date of inspection in the case file and negative traps in control file. The maximum cluster sizes range was again: 200, 300, 400, 500, 600, 700 and 800 m, for larval survey, ovitrap and MosquiTRAP analyses.

### Cluster Reliability

The Reliability of clusters detected by multiple scans using the same dataset was evaluated using Chen methodology [44]. Reliability was estimated for each block (location) of the study area by the equation *Ri* = *Ci*/*S* where *Ri* is the reliability value for location *i*, *S* is the total number of scans performed with a unique dataset, and *Ci* is the number of scans for which the location *i* was within a significant cluster. The reliability measure has a value range from ‘0’ to ‘1,’ where ‘0’ means that the location was not found in a significant cluster in any of the scans and ‘1’ means that the location was within a significant cluster in all scans.

### Evaluation of Space-time Cluster Intersections

To evaluate which monitoring method was best correlated with dengue fever occurrence, space-time intersections were assessed among dengue fever clusters and vector clusters detected using the different datasets: larval survey, ovitrap and MosquiTRAP. Space-time intersection was defined as geographic overlap between dengue fever clusters and vector clusters occurring at same time. The percentage of area and period of intersection was evaluated for each result obtained considering the different ranges of maximum spatial cluster size used.

## Results

### Data Collection

A total of 475 dengue fever cases where registered in the study area, 267 cases on 2007, and 208 cases on the 27 weeks evaluated in 2008 (**Figure 2**). All registered dengue cases were geo-located.

From the total of 1,984 ovitraps used in vector monitoring, 837 were positive (i.e. contained at least one *Aedes sp.* egg when inspected) (**Table 1**). The total number of inspected MosquiTRAPs between the 8^th^ week of 2007 and the 18th week 2008 was 5,774, and 477 of those were positive (i.e. contained at least one *Ae. aegypti* female) (**Table 2**).

The number of larval surveys recorded was 7,247. The LIR*Aa* category registered the largest number of surveys, 5,971, though there was a lower percentage of positivity (i.e. containing *Ae. aegypti* immature forms, 1.41%). From all premises where Culicidae larvae was collected, 82.7% of premises contained *Ae. aegypti* larvae, 3.2% contained *Ae. albopictus* and 30.1% contained other Culicidae species, showing the coexistence of diferent Culicidae species in the study area. The categories SP and SVS recorded 959 and 317 inspected premises respectively, and the percentage of positivity was 2.99% for SP and 47.69% for SVS (**Table 3**). Difference in positivity among the survey categories happened due to the diverging criterion for choosing the premises to be inspected. LIR*Aa* surveys were executed in a sample of premises in a given area and held during a 5-day pre-determined period in the year. In the other categories, SP and SVS, the surveys are conducted in premises presenting certain epidemiological risks. In the SP category, premises considered “strategic location”-those presenting risk conditions to development of *Ae. aegypti-*were inspected biweekly, which increased the probability of finding the vector in these sites. During SVS surveys, premises were inspected for detection of *Ae. aegypti* foci after verification of epidemiologic risk by the surveillance services. Thus, the probability of positivity of SP and SVS surveys was increased relative to LIR*Aa* sampling surveys.

The results showed that there was a different seasonal pattern of egg, larval and adult sampling by ovitrap, larval survey and MosquiTRAP, respectively. The ovitrap was the most sensitive surveillance tool and MosquiTRAP was more sensitive than larval survey. Ovitrap and MosquiTRAP detected the highest infestation rates during periods with higher precipitation and elevated temperatures and higher dengue incidence was verified during vector detection peaks (**Figure 2** **and** **Figure 3**).

### Cluster Detection

In all analyses, clusters of vectors and dengue fever were detected (**Figures S1, S2, S3, S4, S5, S6, S7**). The number of clusters detected was negatively related to MCS radii. The number of dengue fever clusters varied from 16 when MCS was 200 m to 4 when MCS was 800 m. Total cluster area (sum of areas of all the clusters detected using a given MCS) varied from 1.293 km^2^ to 3.692 km^2^, representing 9.36 to 26.74% of study area. The average length of clusters varied from 43.571 (s.d. 24.213) days to 58.750 (s.d. 10.813) days (**Table 4**).

When assessing clusters from vector monitoring, the minimum number of positive ovitrap clusters was 5 and the maximum was 8 clusters. Total cluster area varied from 0.598 km^2^ to 4.339 km^2^, representing from 4.33 to 31.42% of the study area. Positive ovitrap clusters were longer among the monitoring methods with average lengths from 167.00 (s.d. = 81.290) to 258.00 (s.d. = 88.544) days (**Table 5**).

Considering positive MosquiTRAP clusters, the smallest number of detected clusters was 5, and the maximum was 7. Total cluster area varied from 0.465 km^2^ to 5.222 km^2^ representing 3.37 to 37.82% of study area. The average length ranged from 81.833 (s.d. = 32.053) to 95.600 (s.d. = 50.816) days (**Table 6**).

Larval survey detected clusters varied from a total of 3 clusters to 9 detected clusters. Total area of larval survey clusters varied from 0.713 km^2^ to 3.662 km^2^, representing 5.16 to 26.52% of study area. Average lengths ranged from 26.667 (s.d. = 31.628) to 45.667 (s.d. = 20.257) days (**Table 7**).

### Reliability Maps

The development of reliability maps showed the existence of core clusters of the different datasets along the study area (**Figure 4**). Different proportions of core clusters were detected. Considering the blocks forming clusters with the highest *Ri* rates (*Ri* = 0.875), it was verified that 10.80% of the blocks that constituted dengue fever clusters presented this *Ri* value (representing 13.68% of total dengue cluster area). Considering the vector clusters, 12.60% of blocks forming larval survey clusters presented the highest *Ri* value, thus, core cluster blocks represented 6.66% of total clusters area. Blocks with MosquiTRAP positive clusters that had a highest *Ri* value (0.875) represented 1.75% of blocks forming clusters (which was 4.24% of total MosquiTRAP clusters area). Considering ovitrap positive clusters, 1.78% of blocks presented the highest *Ri* value (0.875), representing 10.37% of total ovitrap clusters area (**Table 8**).

### Evaluation of Space-time Cluster Intersections

MosquiTRAP and ovitrap presented temporal and spatial intersections with dengue fever clusters, whereas no spatial or temporal intersection was observed among larval survey and dengue fever clusters at any analysis performed. MosquiTRAP dataset registered 22 space-time intersections to dengue fever clusters, and ovitrap dataset registered 16 space-time intersections (**Figures S1, S2, S3, S4, S5, S6, S7**).

For MosquiTRAP datasets the percentage of spatial intersection with dengue fever clusters varied from 5.38% (MCS = 300 m) to 23.33% (MCS = 600 m). For ovitrap, the spatial intersections varied from 0.97% (MCS = 200 m) to 27.91% (MCS = 800 m) (**Figure 5**).

MosquiTRAP and ovitrap presented similar values of spatial intersection to dengue fever clusters when MCS = 400 m and MCS = 600 m (χ^2^ = 3.588; p = 0.058 and χ^2^ = 0.301; p = 0.584). When considered MCS = 200 m and MCS = 500 m, MosquiTRAP presented greater values of intersection, whereas ovitrap clusters presented greater values at MCS = 300 m (9.30%), 700 m (17.04%) and 800 m (27.91%) (**Figure 5**).

Considering temporal intersections with dengue fever clusters, MosquiTRAP presented values that varied from 5.08% (MCS = 200 m) to 41.36% (MCS = 600 m). Ovitrap temporal intersections values varied from 2.06% (MCS = 200 m) to 64.25% (MCS = 700 m). MosquiTRAP clusters presented higher values of temporal intersection than ovitrap clusters at MCS = 200 m, 400 m, 500 m, and 800 m (**Figure 6**).

## Discussion

Belo Horizonte city presents a humid subtropical climate with two distinct seasons, summer with higher temperatures and high relative humidity, and winter with mild temperatures and low humidity. We found seasonal patterns of dengue fever and vector population size, with a higher risk of dengue during warmer and wetter periods. This confirms the seasonality of dengue that was observed previously by Corrêa and colleagues [6]. It is known that warmer and humid climates enhance the survival of *Ae. aegypti* [50]–[53]. In addition to promoting survival, it is likely that higher temperatures (above 25°C) lead to reduction of extrinsic incubation period and that higher temperatures in conjunction with higher relative humidity increases the bite frequency hence increasing dengue virus transmission [43].

The use of notified dengue fever cases as evidence of pathogen distribution was limited by underreporting that could lead to underestimation of virus circulation and distribution in the areas. In a study conducted at Belo Horizonte municipality, there were 2.5 non-notified dengue fever cases for each case notified by health surveillance service [54]. Therefore, it is possible that non-notified cases could have set up clusters and generated bias. Another potential source of error was the location where transmission occurred; it was assumed that the home address was the infection site. However, since dengue infections are more likely to occur in households, many times affecting multiple hosts in a single house, the assumption of residences as the main infection site is the most realistic approximation [43]. Furthermore, dengue is a disease that usually prevents individuals from undertaking daily activities, resulting in at-home bed rest [55]. Thus, it is likely that infected individuals with viremia remain at home during the infectious period which lasts about 7 days [56]. If the vector was present there was a great probability for transmission to other household members, thus sustaining the viral life cycle. Nevertheless even regarding those difficulties, the use of epidemiologic data for assess dengue pathogen distribution is useful, since a dengue case occurrence mandatorily means vector-host contact.

In the present study, identification of *Aedes* species from eggs sampled at ovitrap was neglected. However, in Belo Horizonte city, collection from larval surveys and sticky traps showed that *Ae. aegypti* is more abundant than other *Aedes* species. The second most abundant *Aedes* species in Belo Horizonte, *Ae. albopictus* still not considered as a dengue vector in Brazil [57], [58], although there are reports of vertical transmission of dengue virus in some Brazilian municipalities [59]–[60].

The use of multiple analyses in SaTScan allowed the identification of core clusters of all datasets in the study area. It was verified in a pre-analysis, that the use of default scaling parameters of maximum clusters sizes unable the interpretation of results, leading to ineffective evaluation of epidemiological data. Hence, using different values of the MCS, it was possible to infer the correlation of vector monitoring and dengue transmission from smaller local scales.

Monitoring the vector by ovitrap and MosquiTRAP showed temporal and spatial overlap with dengue fever cases, both presenting similar results. Nevertheless, MosquiTRAP space-time intersections to dengue fever clusters occurred in greater numbers than ovitrap ones. Larval survey registries showed no correlation to dengue cases occurrence (**Figure 5** **,** **Figure 6** **and Figures S1, S2, S3, S4, S5, S6, S7**).

Comparing ovitrap and MosquiTRAP clusters, it was observed that positive oviposition traps clusters presented longer duration periods, that averaged between 167 (s.d. 81.29) days to 258 (s.d. 88.54) days, while MosquiTRAP positive clusters duration ranged from 81.83 (s.d. 32.05) days to 95.60 (s.d. 50.82) days (**Table 5** and **Table 6**). Thus, considering the clusters duration periods, it was more probable that ovitrap clusters intersected dengue fever clusters than MosquiTRAP ones. Nevertheless using this long durating ovitrap clusters for estimative of dengue transmissions risks may lead a overestimating risk assessement, since as demonstrated, the detection of risk areas with *Aedes sp.* during most of the study period by ovitrap method does not necessarily translated to dengue fever occurrence.

For better evaluation of the temporal correlation between dengue fever clusters and monitoring methods, the precedence of vector clusters to dengue fever clusters was evaluated, being estimated the average days that a vector cluster preceded a spatially overlapped dengue fever cluster. It was found that ovitrap clusters occurred earlier than the MosquiTRAP clusters, showing that the MosquiTRAP method more accurately estimated the timing of dengue fever clusters. The ovitraps Clusters occurred between 40 to 283 (s.d. 168.40) days before dengue fever cases, while MosquiTRAP clusters ranged from 65.50 (s.d. 58.69) to 94 (s.d. 14.30) days prior to cases (**Figure 7**; **Table 9** & **Table 10**). A major point concerning vector monithoring tools is that precise methods for identifying when critical areas will undergo high rates of transmission is the key for allocation of limited resources and deployment of effective methods for vector control.

Studies comparing the sensitivity of ovitrap and MosquiTRAP showed that the sensitivity of the oviposition trap is higher [20], suggesting that ovitraps are effective for detecting low level infestations. However, the indices cannot be used for estimating the abundance of adults [61]. Our results demonstrates that detection of female *Ae. aegypti* by MosquiTRAP provided a good correlation to occurrence of dengue. As stated by Fávaro and colleagues [20], the greater precision that MosquiTRAP provides for capturing adults allows better estimation of the adult population. This justifies the use of the trap in control programs, since its entomological indices permits assessments of risk and evaluation of vector control measures more precisely, and allows reliable determination of thresholds for the occurrence of dengue transmission in a given area. However, it is important to consider that the correlation between the occurrence of dengue fever cases and vector detection only occurs if the dynamics between the vector, the virus and hosts are favorable, which depends on several factors other than the population density of *Ae. aegypti* [62]. Another point to consider is that the installation and inspection of ovitraps occurred every two weeks, while MosquiTRAPs were visited weekly and it was not possible to infer if cluster detection was affected by these different frequencies of trap installation and inspection in the study area. Another point to consider is the limitations of the circular scan technique, that may overestimate cluster area or may omit real cluster regions if real clusters are irregular shaped. For overcoming this type of limitation, it is possible to use arbitrary geometry scan techniques that permit detection of more geographically precise clusters [63].

The seasonal pattern of dengue fever and vector presence throughout the study period indicated that the risk of disease burden was continuous in these areas. In Belo Horizonte city, vector control is executed according to the PNCD [10], and premise inspections for *Ae. aegypti* foci are held bimonthly. The most common breeding sites are likely to consist of small artificial reservoirs exposed to rainfall. Data from Belo Horizonte entomologic surveillance shows that in rainy seasons the main breeding sites are disposable recipients, whereas in times of low rainfall, the breeding sites are most often potable water reservoirs [15]. Vector control becomes difficult across seasons due to the great diversity of breeding sites, which allow for the existence of populations that escape inspection during routine vector control activities. Thus, the *Ae. aegypti* skip oviposition behavior, with distribution of small amounts of eggs in several different reservoirs, may contribute to the maintenance of infestations even after execution of inspections, since the complete elimination of water containers or containers that posteriorly will be water filled, is unlikely. The replacement of receptacles in the environments at a higher frequency than the frequency of surveys held by vector control personnel, reduces control efficacy. In these situations, the use of larvicides with residual action is insufficient to affect vector occurrence and distribution.

Considering the presence of vector populations throughout the study period, it is likely that the control methodology based on routine research for larval destruction and use of larvicides, did not affect dengue epidemiological indices in the area. Thus, additional control efforts should be based on data supplied by entomological surveillance, defining priority areas for implementation of vector control activities. It is therefore necessary to use surveillance tools capable of providing accurate entomological data in order to determine dengue transmission risk based on *Ae. aegypti* presence registries.

We conclude that MosquiTRAP presented greater correlation to dengue fever distribution, both temporally and spatially relative to larval survey and ovitrap. Moreover, to improve vector control actions, it is necessary to use surveillance tools capable of fine scale assessment of dengue transmission risks.

## Supporting Information

Figure S1 **Summary of clusters intersections detected considering 200** **m of Maximum Cluster Size (MCS).** All significant clusters detected using 200 m as the MCS value are displayed for each monitoring method. Dengue fever clusters (red), positive MosquiTRAP clusters (dark green), positive ovitrap clusters (blue) and positive larval survey clusters (yellow). The spatial and temporal overlap between dengue fever clusters and vector clusters are represented in black.
(TIF)

Figure S2 **Summary of clusters intersections detected considering 300** **m of Maximum Cluster Size (MCS).** All significant clusters detected using 300 m as the MCS value are displayed for each monitoring method. Dengue fever clusters (red), positive MosquiTRAP clusters (dark green), positive ovitrap clusters (blue) and positive larval survey clusters (yellow). The spatial and temporal overlap between dengue fever clusters and vector clusters are represented in black.
(TIF)

Figure S3 **Summary of clusters intersections detected considering 400** **m of Maximum Cluster Size (MCS).** All significant clusters detected using 400 m as the MCS value are displayed for each monitoring method. Dengue fever clusters (red), positive MosquiTRAP clusters (dark green), positive ovitrap clusters (blue) and positive larval survey clusters (yellow). The spatial and temporal overlap between dengue fever clusters and vector clusters are represented in black.
(TIF)

Figure S4 **Summary of clusters intersections detected considering 500** **m of Maximum Cluster Size (MCS).** All significant clusters detected using 500 m as the MCS value are displayed for each monitoring method. Dengue fever clusters (red), positive MosquiTRAP clusters (dark green), positive ovitrap clusters (blue) and positive larval survey clusters (yellow). The spatial and temporal overlap between dengue fever clusters and vector clusters are represented in black.
(TIF)

Figure S5 **Summary of clusters intersections detected considering 600** **m of Maximum Cluster Size (MCS).** All significant clusters detected using 600 m as the MCS value are displayed for each monitoring method. Dengue fever clusters (red), positive MosquiTRAP clusters (dark green), positive ovitrap clusters (blue) and positive larval survey clusters (yellow). The spatial and temporal overlap between dengue fever clusters and vector clusters are represented in black.
(TIF)

Figure S6 **Summary of clusters intersections detected considering 700** **m of Maximum Cluster Size (MCS).** All significant clusters detected using 700 m as the MCS value are displayed for each monitoring method. Dengue fever clusters (red), positive MosquiTRAP clusters (dark green), positive ovitrap clusters (blue) and positive larval survey clusters (yellow). The spatial and temporal overlap between dengue fever clusters and vector clusters are represented in black.
(TIF)

Figure S7 **Summary of clusters intersections detected considering 800** **m of Maximum Cluster Size (MCS).** All significant clusters detected using 800 m as the MCS value are displayed for each monitoring method. Dengue fever clusters (red), positive MosquiTRAP clusters (dark green), positive ovitrap clusters (blue) and positive larval survey clusters (yellow). The spatial and temporal overlap between dengue fever clusters and vector clusters are represented in black.
(TIF)
