# Supplementary figures and images for: Dengue Fever Occurrence and Vector Detection by Larval Survey, Ovitrap and MosquiTRAP: A Space-Time Clusters Analysis
Source: PLoS One. 2012 Jul 25;7(7):e42125. doi: 10.1371/journal.pone.0042125 (PMC3405049; doi:10.1371/journal.pone.0042125)

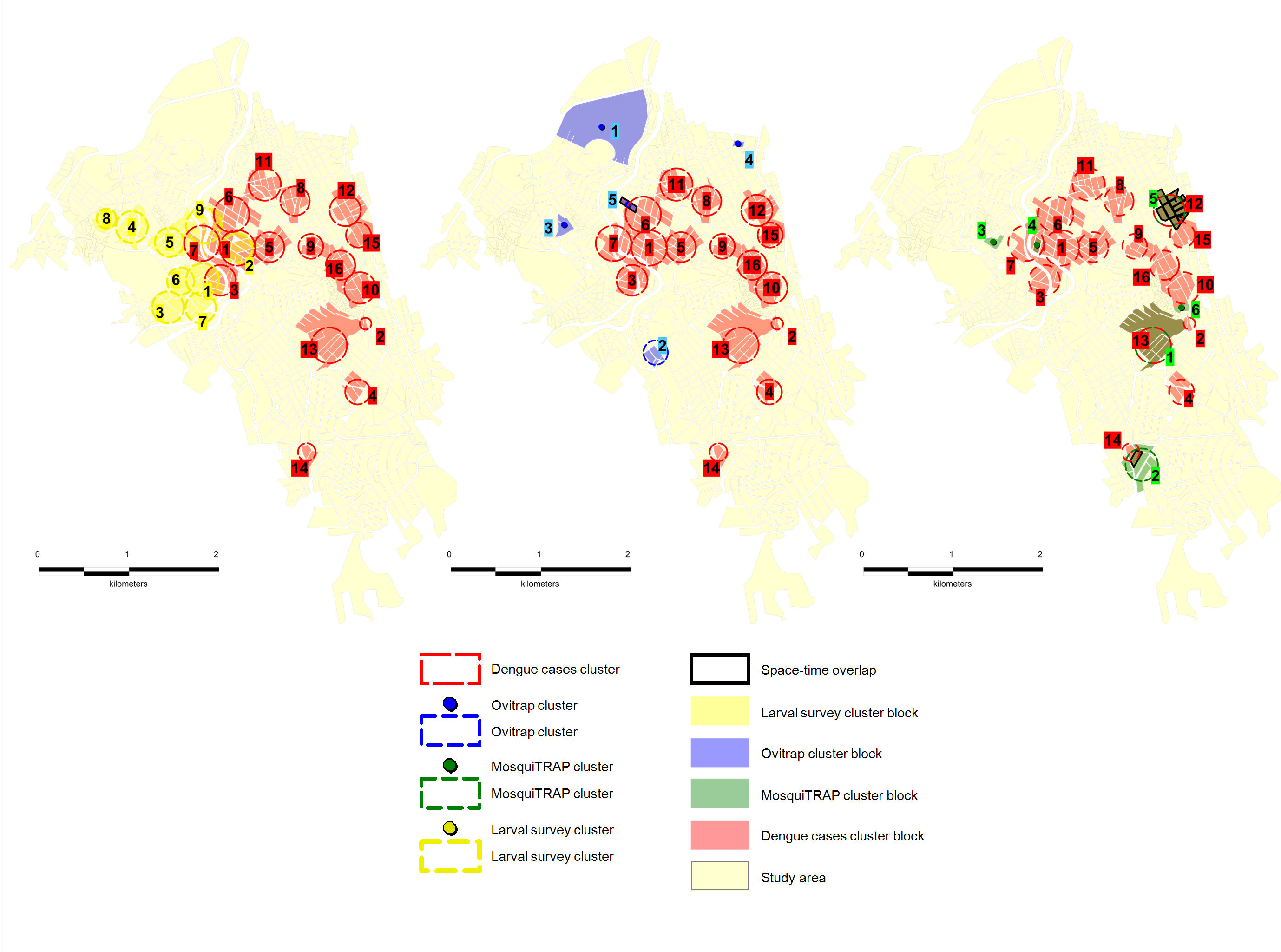

Supplement: Figure S1 — Summary of clusters intersections detected considering 200 m of Maximum Cluster Size (MCS). All significant clusters detected using 200 m as the MCS value are displayed for each monitoring method. Dengue fever clusters (red), positive MosquiTRAP clusters (dark green), positive ovitrap clusters (blue) and positive larval survey clusters (yellow). The spatial and temporal overlap between dengue fever clusters and vector clusters are represented in black. (TIF) [file pone.0042125.s001.tif]

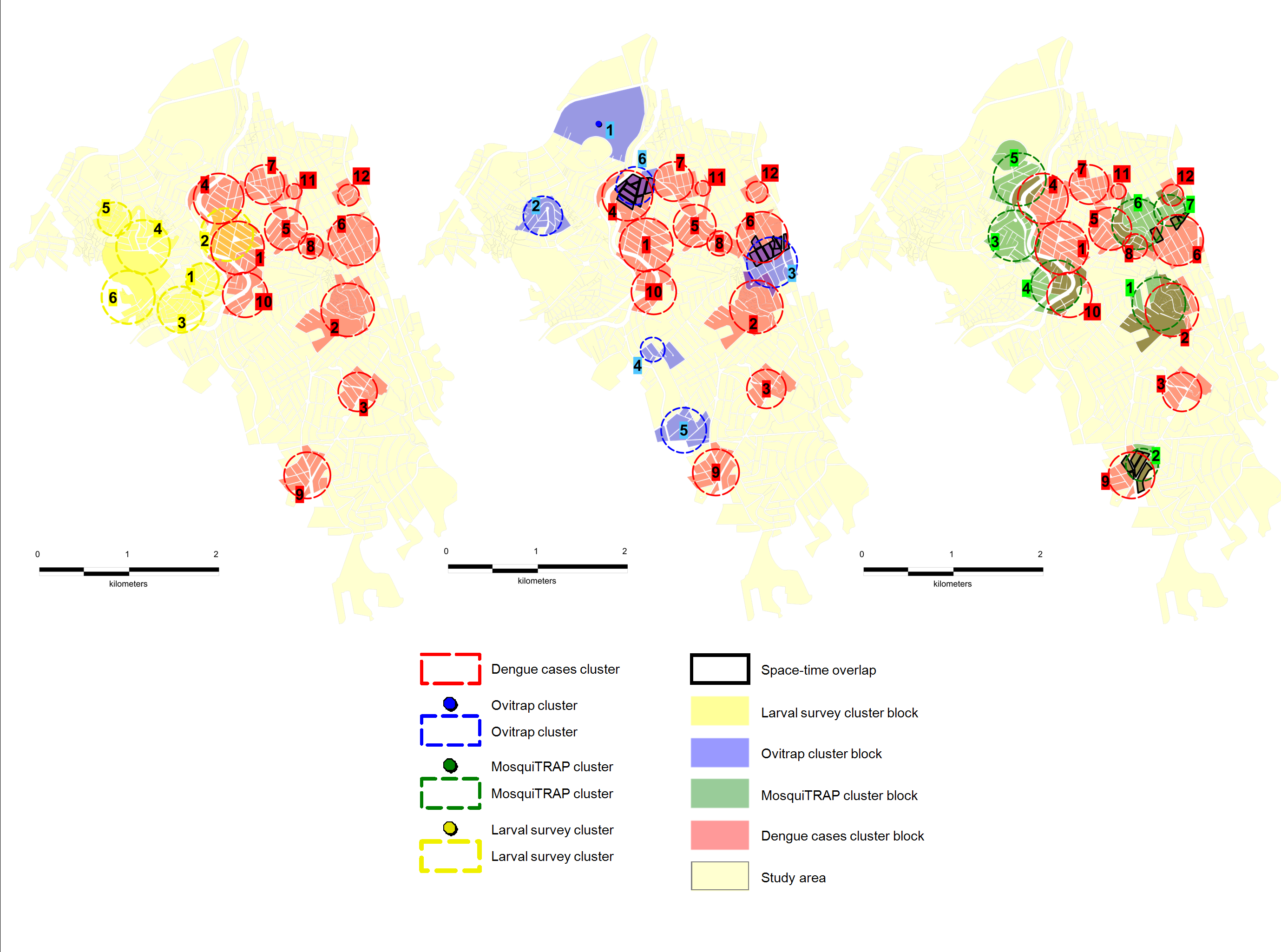

Supplement: Figure S2 — Summary of clusters intersections detected considering 300 m of Maximum Cluster Size (MCS). All significant clusters detected using 300 m as the MCS value are displayed for each monitoring method. Dengue fever clusters (red), positive MosquiTRAP clusters (dark green), positive ovitrap clusters (blue) and positive larval survey clusters (yellow). The spatial and temporal overlap between dengue fever clusters and vector clusters are represented in black. (TIF) [file pone.0042125.s002.tif]

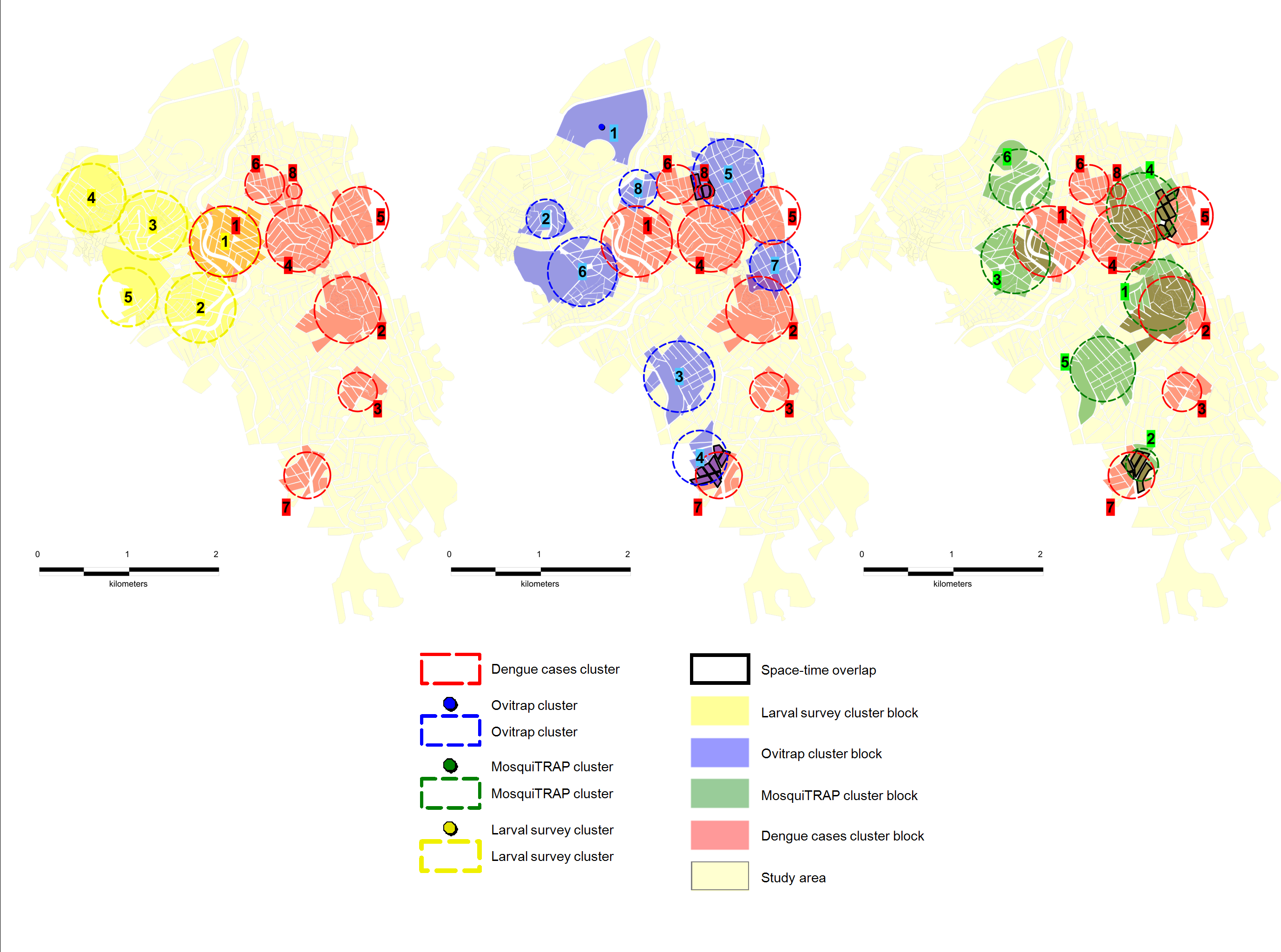

Supplement: Figure S3 — Summary of clusters intersections detected considering 400 m of Maximum Cluster Size (MCS). All significant clusters detected using 400 m as the MCS value are displayed for each monitoring method. Dengue fever clusters (red), positive MosquiTRAP clusters (dark green), positive ovitrap clusters (blue) and positive larval survey clusters (yellow). The spatial and temporal overlap between dengue fever clusters and vector clusters are represented in black. (TIF) [file pone.0042125.s003.tif]

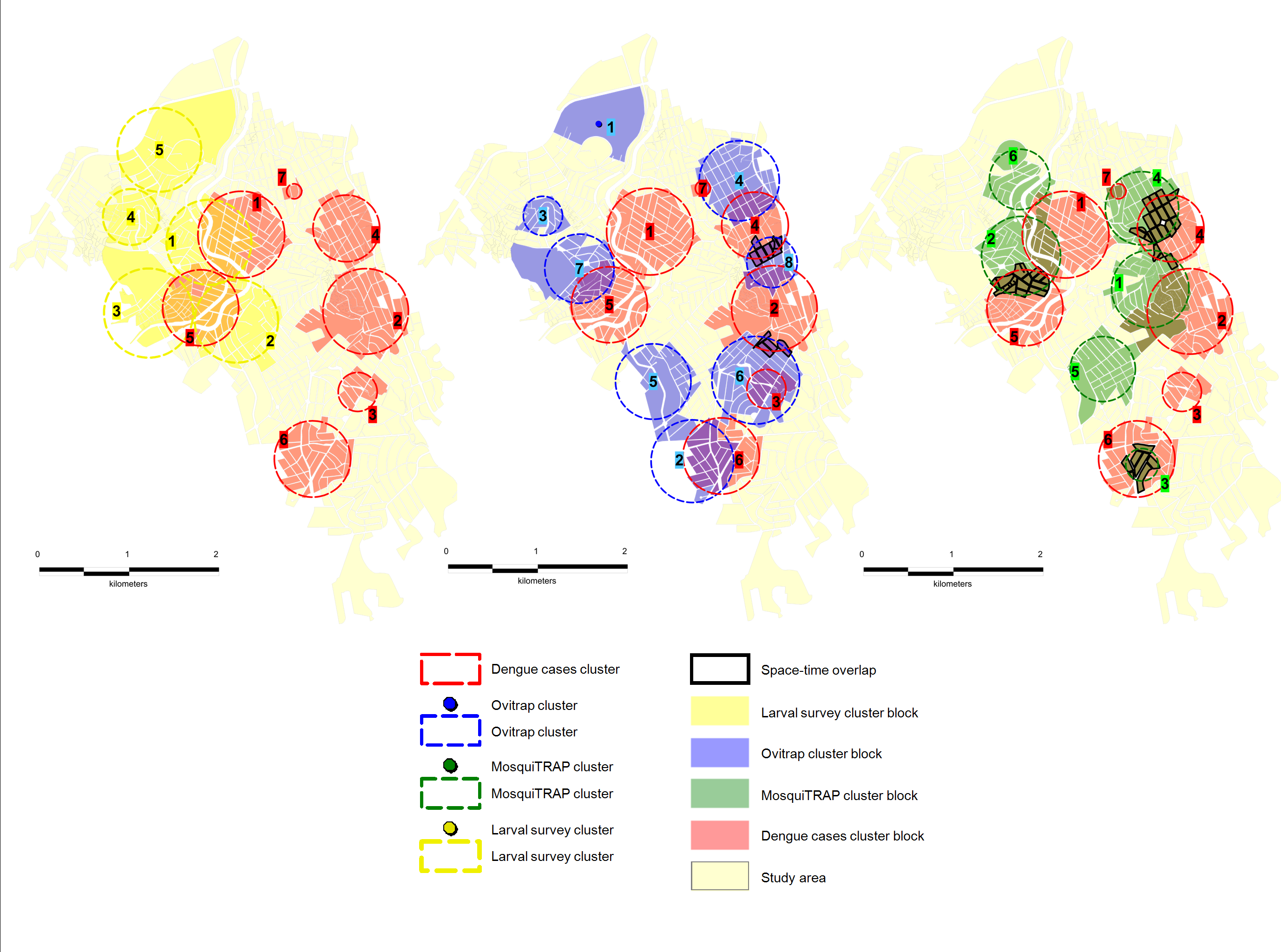

Supplement: Figure S4 — Summary of clusters intersections detected considering 500 m of Maximum Cluster Size (MCS). All significant clusters detected using 500 m as the MCS value are displayed for each monitoring method. Dengue fever clusters (red), positive MosquiTRAP clusters (dark green), positive ovitrap clusters (blue) and positive larval survey clusters (yellow). The spatial and temporal overlap between dengue fever clusters and vector clusters are represented in black. (TIF) [file pone.0042125.s004.tif]

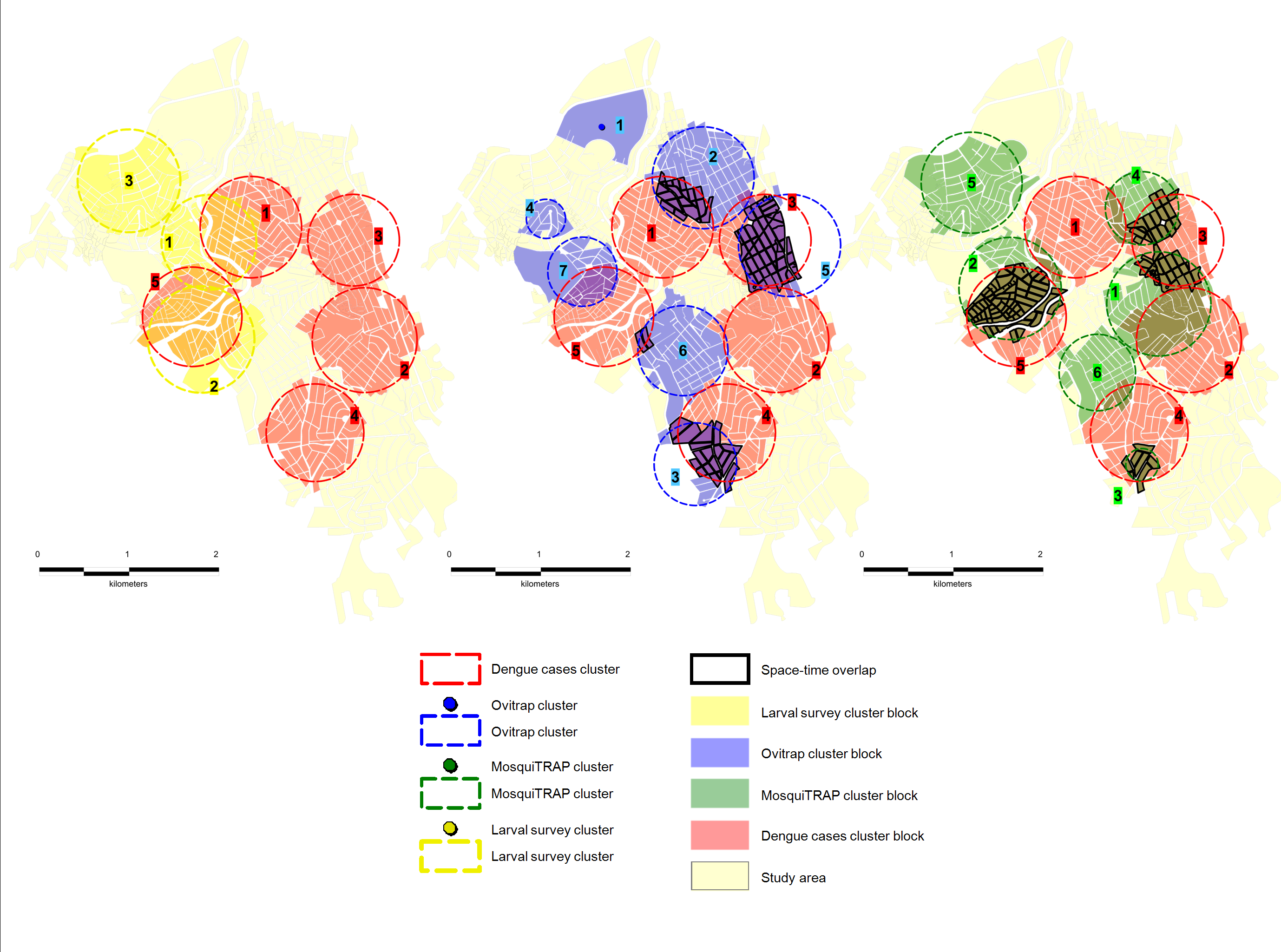

Supplement: Figure S5 — Summary of clusters intersections detected considering 600 m of Maximum Cluster Size (MCS). All significant clusters detected using 600 m as the MCS value are displayed for each monitoring method. Dengue fever clusters (red), positive MosquiTRAP clusters (dark green), positive ovitrap clusters (blue) and positive larval survey clusters (yellow). The spatial and temporal overlap between dengue fever clusters and vector clusters are represented in black. (TIF) [file pone.0042125.s005.tif]

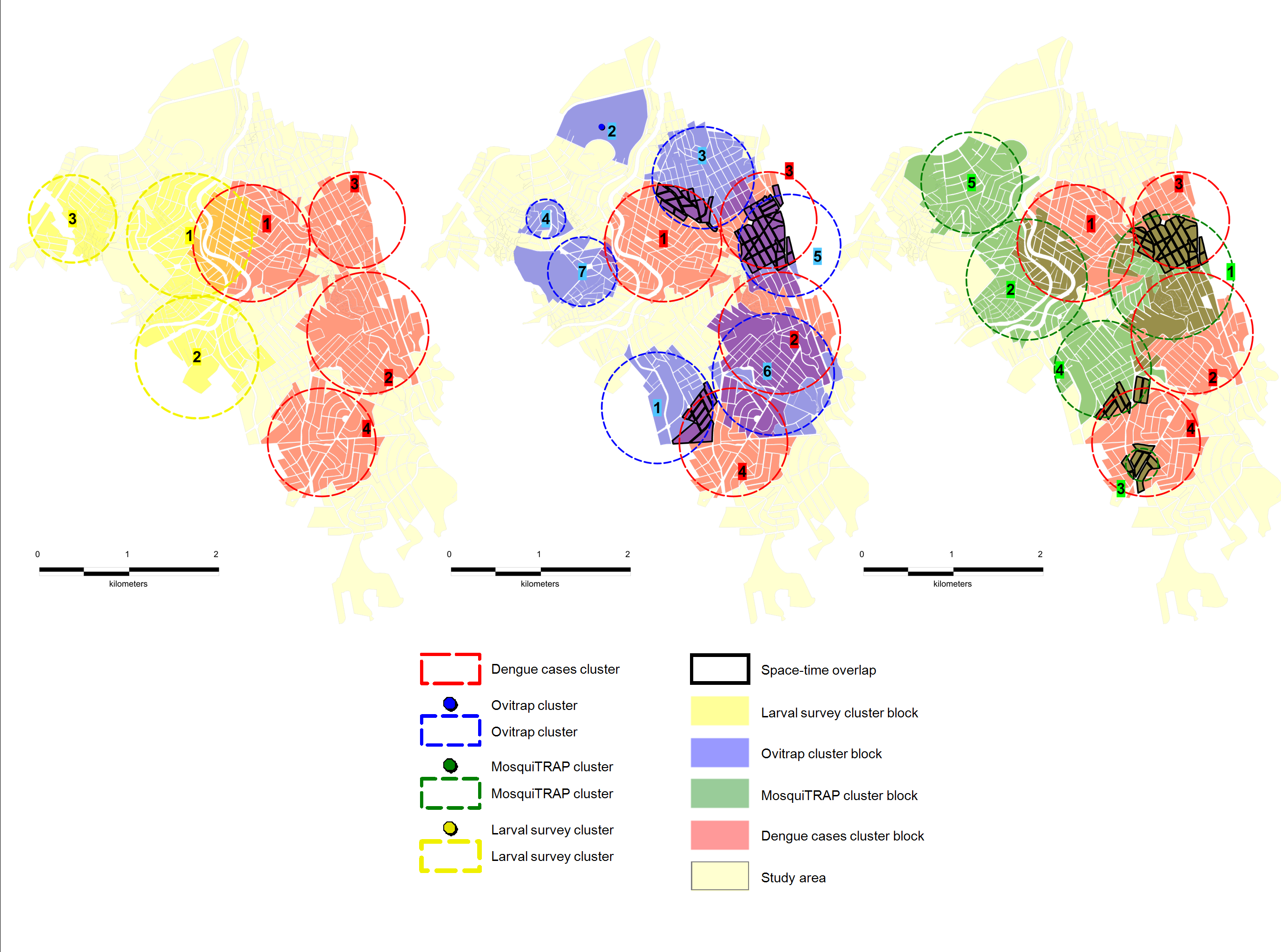

Supplement: Figure S6 — Summary of clusters intersections detected considering 700 m of Maximum Cluster Size (MCS). All significant clusters detected using 700 m as the MCS value are displayed for each monitoring method. Dengue fever clusters (red), positive MosquiTRAP clusters (dark green), positive ovitrap clusters (blue) and positive larval survey clusters (yellow). The spatial and temporal overlap between dengue fever clusters and vector clusters are represented in black. (TIF) [file pone.0042125.s006.tif]

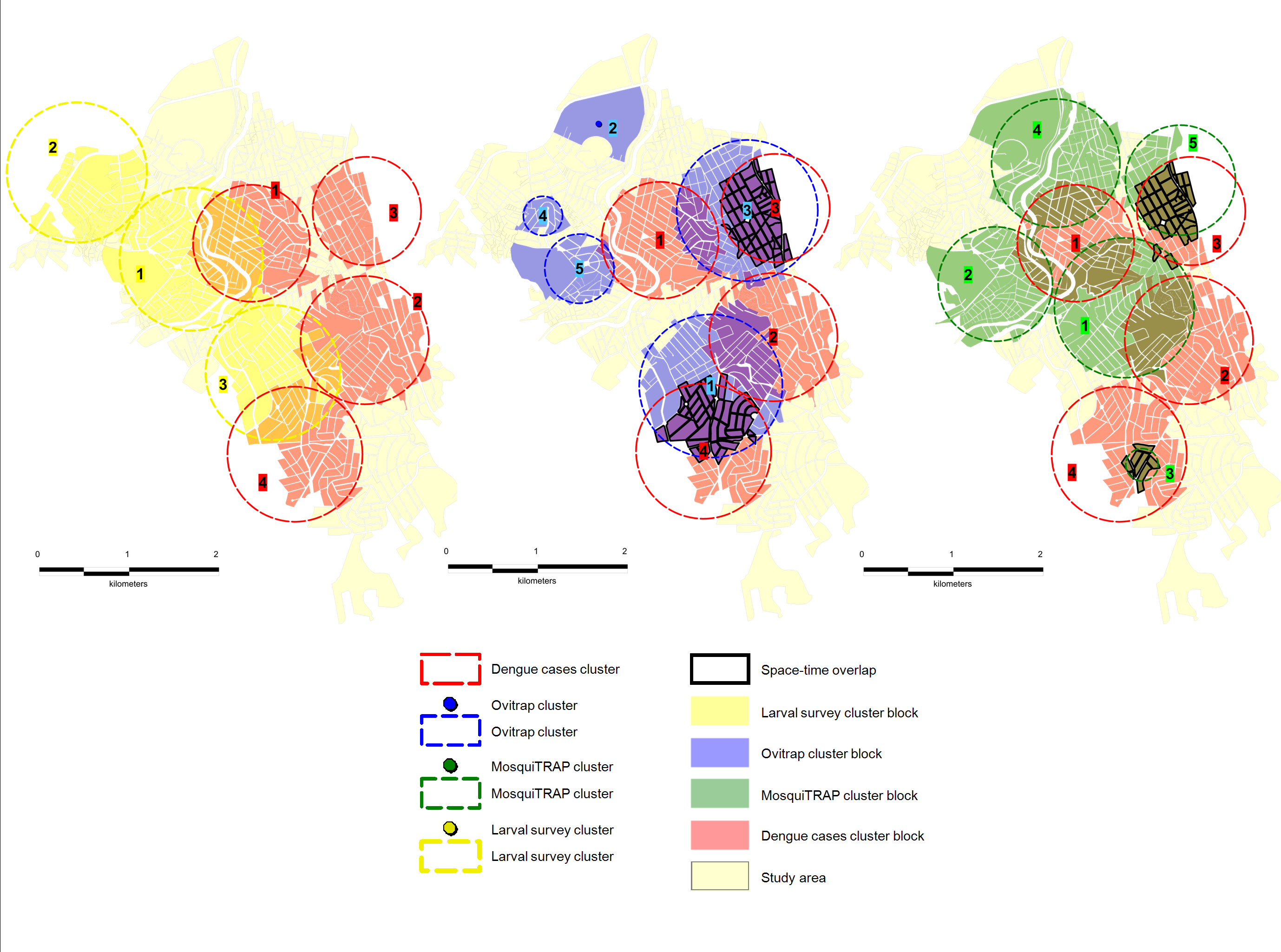

Supplement: Figure S7 — Summary of clusters intersections detected considering 800 m of Maximum Cluster Size (MCS). All significant clusters detected using 800 m as the MCS value are displayed for each monitoring method. Dengue fever clusters (red), positive MosquiTRAP clusters (dark green), positive ovitrap clusters (blue) and positive larval survey clusters (yellow). The spatial and temporal overlap between dengue fever clusters and vector clusters are represented in black. (TIF) [file pone.0042125.s007.tif]
